# Secretory Products of the Human GI Tract Microbiome and Their Potential Impact on Alzheimer's Disease (AD): Detection of Lipopolysaccharide (LPS) in AD Hippocampus

**DOI:** 10.3389/fcimb.2017.00318

**Published:** 2017-07-11

**Authors:** Yuhai Zhao, Vivian Jaber, Walter J. Lukiw

**Affiliations:** ^1^LSU Neuroscience Center, Louisiana State University Health Science Center New Orleans, LA, United States; ^2^Department of Anatomy and Cell Biology, Louisiana State University Health Science Center New Orleans, LA, United States; ^3^Department of Ophthalmology, Louisiana State University Health Science Center New Orleans, LA, United States; ^4^Department of Neurology, Louisiana State University Health Science Center New Orleans, LA, United States

**Keywords:** 42 amino acid amyloid-beta (Aβ42) peptide, Alzheimer's disease (AD), *Bacteriodetes fragilis* (*B. fragilis*), *Escherichia coli* (*E. coli*), lipopolysaccharide (LPS), microbiome, small non-coding RNAs (sncRNAs), thanatomicrobiome

## Abstract

Although the potential contribution of the human gastrointestinal (GI) tract microbiome to human health, aging, and disease is becoming increasingly acknowledged, the molecular mechanics and signaling pathways of just how this is accomplished is not well-understood. Major bacterial species of the GI tract, such as the abundant Gram-negative bacilli *Bacteroides fragilis* (*B. fragilis*) and *Escherichia coli* (*E. coli*), secrete a remarkably complex array of pro-inflammatory neurotoxins which, when released from the confines of the healthy GI tract, are pathogenic and highly detrimental to the homeostatic function of neurons in the central nervous system (CNS). For the first time here we report the presence of bacterial lipopolysaccharide (LPS) in brain lysates from the hippocampus and superior temporal lobe neocortex of Alzheimer's disease (AD) brains. Mean LPS levels varied from two-fold increases in the neocortex to three-fold increases in the hippocampus, AD over age-matched controls, however some samples from advanced AD hippocampal cases exhibited up to a 26-fold increase in LPS over age-matched controls. This “Perspectives” paper will further highlight some very recent research on GI tract microbiome signaling to the human CNS, and will update current findings that implicate GI tract microbiome-derived LPS as an important internal contributor to inflammatory degeneration in the CNS.

## Introduction: the human GI tract microbiome

The human GI tract is fundamentally a highly vascularized and extensively innervated, columnar epithelial-cell lined tube about 9 m (30 feet) in length that consists of the stomach, small intestine (duodenum, jejunum, and ileum) and large intestine (cecum, colon, rectum, and anal canal; Reinus and Simon, 2014). Each anatomical region of this tubular structure harbors a complex and dynamic microbiome, containing ~1,000 different species of anaerobic or facultative anaerobic bacteria that appear to be characteristic for that GI tract segment. Indeed, the dynamism of the GI tract microbiome along its length is in part reflected by the abundance, speciation, complexity and stoichiometry of individual resident bacterial species. In addition to the major bacterial component of the GI tract are microbial eukaryotes, archaea, fungi, protozoa, viruses, and other commensal microorganisms which make up the remainder. Together with host cells these jointly comprise the complete metaorganism: (i) whose symbiotic associations and interactions are indispensable for homeostatic physiological functions in human health; and (ii) which exhibit alterations in composition in response to dietary factors, developmental stage, GI tract disturbances, aging, and neurological disorders, including AD (Bhattacharjee and Lukiw, 2013; Hill et al., 2014; Perez et al., 2014; Potgieter et al., 2015; Zhao and Lukiw, 2015; Alkasir et al., 2016; Ghaisas et al., 2016; Hu et al., 2016; Lukiw, 2016; Pistollato et al., 2016; Scheperjans, 2016).

## GI tract bacterial microbiome—exudates and secretory products

Two large prokaryotic classes of Bacteria (*or “Eubacteria”*) and Archaea (or “*Archaeobacteria”*) have been recently reclassified (as of 10/2016) into 35 phyla (http://www.bacterio.net/-classifphyla.html) or major bacterial divisions. Interestingly the GI tract microbiome of *Homo sapiens* has co-evolved with just two major phyla: *Bacteriodetes*, which make up ~20% of all GI tract bacteria, and *Firmicutes*, which make up ~80% of all GI tract bacteria; with *Actinobacteri*a (~3%), *Proteobacteria* (~1%), and *Verrumicrobia* (~0.1%) making up significantly smaller fractions. These five bacterial groups appear to constitute the essential “*core*” of the human GI tract microbiome (http://www.bacterio.net/-classifphyla.html; Zhao et al., 2015; Hug et al., 2016; Lloyd-Price et al., 2016; Sender et al., 2016). The vast proportion of all GI tract microbiota consists of anaerobic or facultative anaerobic bacteria (Bhattacharjee and Lukiw, 2013; Heintz and Mair, 2014; Köhler et al., 2016; Lloyd-Price et al., 2016). For example, although variable, the obligate anaerobe *Bacteroides fragilis* (*B. fragilis*; phyla *Bacteroidetes)* and the facultative anaerobe *Escherichia coli* (*E. coli*; phyla Proteobacteria): (i) together constitute ~35–40 percent of all GI tract bacteria; (ii) are the most abundant Gram-negative bacilli of the middle and lower colon, respectively, of the human GI tract; and (iii) constitute about ~30–50 percent of the dry weight of fecal matter. *B. fragilis* or *E. coli* require about 20 min to divide under optimal conditions of commensal bacterial growth, and unless special biophysical processes of growth dynamics are in operation (such as dormancy, hibernation, spore formation, etc.) have a life span of up to several hours (Choi and Cho, 2016; Pinti et al., 2016; Todar, 2016). Interestingly, species of the obligate anaerobe *Bacteroides* such as *B. fragilis* display remarkably diverse antibiotic resistance mechanisms and exhibit the highest resistance rates of any anaerobic pathogen. This includes an inherent high-level resistance to penicillin through their ability to produce beta-lactamase enzymes which endow them with multiple resistance to β-lactam antibiotics such as penicillin and cephamycin (Ayala et al., 2005; Bush and Bradford, 2016; Hu et al., 2016). Specific species of *Bacteroidetes* such as *Bacteroides fragilis* (*B. fragilis*), normally an abundant commensal microorganism of the middle GI tract, are known to be generally beneficial to human health through their ability to digest dietary fiber and related dietary fiber precursors containing substances such as cellulose, lignin, and pectin, which are normally resistant to the action of host digestive enzymes.

**Figure 1:**
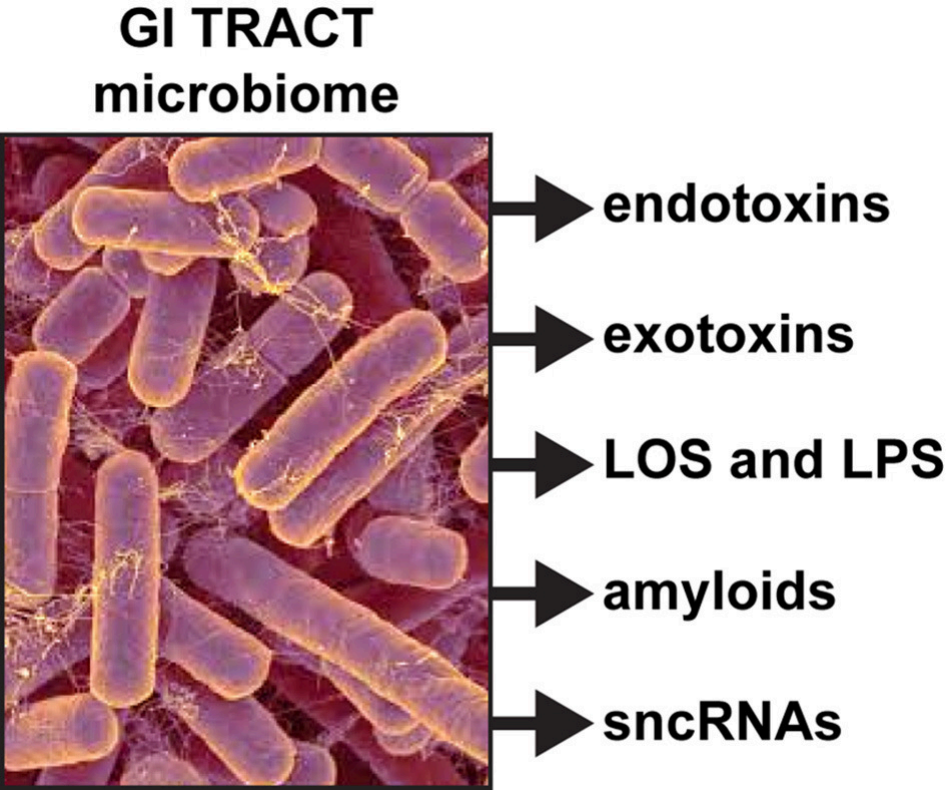
Like other Gram-negative bacilli, the gastrointestinal (GI) tract abundant *Bacteroides fragilis* (micrograph of *B. fragilis* shown; original photo courtesy of Rosa Rubicondior; (http://rosarubicondior.blogspot.com/2014/11/evolving-cooperation-but-for-who-or-what.html) is capable, when stressed, of releasing a broad spectrum of highly neurotoxic, pro-inflammatory and potentially pathogenic molecules; these comprise five major classes of secreted molecules and include endotoxins, exotoxins, lipooligosacahride (LOS) and lipopolysaccharide (LPS), amyloids, and small non-coding RNAs (sncRNA). For example, the human GI tract-abundant *B. fragilis* secretes the endotoxin fragilysin and *B. fragilis* LPS (BF-LPS) both of which have been shown recently to be strongly pro-inflammatory and extremely neurotoxic toward human CNS neurons in primary culture (Li et al., 2016; Lukiw, 2016). While the phyla *Bacteriodetes* (~20% of all GI tract bacteria), *Firmicutes* (~80% of all GI tract bacteria), *Actinobacteria, Proteobacteria*, and *Verrumicrobia* (together, typically ~4% of all GI tract bacteria), are the most common microbes in the human GI tract microbiome it should be kept in mind that other microbes including fungus, protozoa, viruses, and other commensal microorganisms may also contribute neurotoxic exudates which are highly toxic and detrimental to the homeostasis of CNS neurons.

Dietary fibers are catabolized into digestible short-chain fatty acids (SCFAs), volatile fatty acids and polysaccharides in part through the biosynthetic capability of this GI tract abundant bacillus (Keenan et al., 2016; Scheperjans, 2016). When *B. fragilis* escapes the highly compartmentalized microbe-dense environment of the GI tract (10^11^ microbes per gram of fecal matter), they can induce substantial systemic inflammatory pathology with significant sickness, morbidity and mortality (Choi et al., 2016; Fathi and Wu, 2016; Cattaneo et al., 2017; Shivaji, 2017). Enterotoxigenic strains of *B. fragilis* have been associated with bacteremia, colitis, diarrhea, sepsis, systemic infection, and the development of GI tract cancers and neurological disorders, including AD, that have an increased incidence with aging (Choi et al., 2016; Fathi and Wu, 2016; Keenan et al., 2016; Scheperjans, 2016). Interestingly, certain species of Bacteroidetes have been recently shown to propagate in animal models fed high fat-cholesterol (HFC) diets deprived of sufficient intake of dietary fiber; this suggests that sufficient dietary fiber may have a significant role in regulating the abundance, complexity and stoichiometry of certain species in the GI tract microbiome, including *B. fragilis* (Heinritz et al., 2016; Köhler et al., 2016; Pistollato et al., 2016; unpublished observations). In addition to these positive health benefits however, these vast numbers of human GI tract resident Gram-negative bacilli when stressed secrete prodigious quantities of endotoxins, exotoxins, endotoxins, exotoxins, lipooligosacahrides (LOSs) and lipopolysaccharides (LPSs), amyloids, and small non-coding RNAs (sncRNAs; see below and Figure 1).

## Endotoxins and exotoxins

Generally, microbiome-derived endotoxins are heat-stable polypeptides associated with the outer membranes of the cell wall of Gram-negative bacteria. They may be composed in part by the Lipid A component of LPS, and once they diffuse into the local environment induce irritation of the GI tract epithelia, capillaries and blood vessels inducing hemorrhage and various pro-inflammatory effects. Endotoxins also induce fever, hemorrhagic shock, diarrhea, altered resistance to bacterial infection, leukopenia followed by leukocytosis, and numerous other systemic effects (Choi et al., 2016; Seong et al., 2016; Zhan and Davies, 2016). For example, in addition to their prodigious LPS generation (see below), *B. fragilis* endotoxins are a leading cause of anaerobic bacteremia, sepsis and systemic inflammatory distress through their generation of the highly pro-inflammatory zinc metalloproteinase fragilysin, also known as *B. fragilis* toxin or BFT (Zhao and Lukiw, 2015; Choi et al., 2016; Fathi and Wu, 2016). BFT has recently been shown to effectively disrupt epithelial cells of GI tract barriers via cleavage of the synaptic type-1 transmembrane zonula adhesion calcium-dependent adhesion protein E-cadherin (Choi et al., 2016; Seong et al., 2016; Zhan and Davies, 2016). It is currently not understood if GI tract- or BBB-disrupting proteolytic endotoxins such as BFT are able to propagate their pathogenic activities via the systemic circulation to further disrupt the GI tract or BBB at distant sites, to ultimately transfer endotoxins, exotoxins, LPSs, amyloids and/or sncRNAs into the cerebrovascular circulation to target brain cells within the CNS. *B. fragilis* has been suggested to contribute to neurodevelopmental pathology in autism spectrum disorder (ASD; Hsiao et al., 2013; Hofer, 2014; Keaney and Campbell, 2015). It has also recently been reported that along with BFTs amyloid peptide-dependent changes in synaptic adhesion affect both the function and integrity of synapses, suggesting that the observed failure of synaptic adhesion in AD play key roles in the progressive disruption of functional signaling throughout neuronal networks, as is observed in AD brain (Lin et al., 2014; Seong et al., 2015; Leshchyns'ka and Sytnyk, 2016).

Exotoxins are generally complex soluble polypeptides produced on the inside of pathogenic bacteria as part of their normal growth and metabolism, and these are typically excreted by living cells or released during bacterial cell lysis into the surrounding medium. The relatively short lifespan of GI tract bacteria (see above) and their subsequent lysis indicate that lysed bacteria contents may be a relatively persistent source of exotoxins which may need to be either efficiently neutralized or eliminated by the GI tract. Interestingly, under some conditions in rodents certain endotoxins are so toxic that they may be lethal to the host before the innate immune system has a chance to mount immune defenses to promote their neutralization (Bhattacharjee and Lukiw, 2013; Asti and Gioglio, 2014; Hill et al., 2014; Hill and Lukiw, 2015).

## Lipooligosacahride (LOS) and lipopolysaccharide (LPS)

As an abundant Gram negative bacilli of the human GI tract microbiome both *B. fragilis* and *E. coli* secrete lipooligosacahrides (LOS) and lipopolysaccharides (LPS) that are strongly immunogenic and highly pro-inflammatory toward human neurons (Bian et al., 2011; Alkasir et al., 2016; Fathi and Wu, 2016; Foster et al., 2016; Ghaisas et al., 2016; Hug et al., 2016; Lukiw, 2016; Rogers and Aronoff, 2016; Sender et al., 2016; Sharon et al., 2016). LPSs, as characteristic components of the outer leaflet of the outer membrane of Gram-negative bacteria shed into the extracellular space, play key roles in host-pathogen interactions and the innate-immune system (Hill and Lukiw, 2015; Zhao et al., 2015; Maldonado et al., 2016). While LPSs contain large and hypervariable oligosaccharide/polysaccharide regions, the relatively conserved lipid region (lipid A) is the endotoxic and biologically active moiety that is largely responsible for septic shock (Jiang et al., 2016; Maldonado et al., 2016). A canonic LPS structure is represented by that of *E. coli* LPS, one of the most potent neurotoxic lipid A species known, consisting of a 1,4′-biphosphorylated glucosamine disaccharide bearing six fatty acids which are unbranched chains 12–14 methyl(ene) units in length. Other “lipid A” species show variability in the number, length, and composition of the attached fatty acids, as well as variability in the degree of phosphorylation and number and types of substituted phosphate ligands. For instance, BF-LPS lipid A is penta-acylated and mono-phosphorylated, and contains branched fatty acids 15–17 methyl(ene) units in length; deviations from the canonical lipid A structure are known to have a profound impact on innate-immune responses. Gram-negative bacterial exudates such as BF-LPSs are hypervariable in composition, and different *Bacteroidetes* species appear to generate unique temporal patterns of LPS production. These exhibit rapid and remarkably adaptive changes in LPS structure and alterations in *damage- or pathogen-associated molecular patterns (*DAMP/PAMP) as strategies for host immune evasion (Friedland, 2015; Land, 2015; Maldonado et al., 2016; Richards et al., 2016). Here, for the first time, we provide evidence that *E. coli* LPS is abundant in neocortical and hippocampal extracts from AD brain, regions of the human limbic system targeted by intense neuro-inflammation characteristic of the AD process (see Figure 1 and legend). Similarly the pathological actions of LPS on the induction of pro-inflammatory signaling in primary human neurons have recently been demonstrated, and additional studies are in progress (Lukiw, 2016).

## Amyloids

Atypical amyloid generation, aggregation, folding, and impaired clearance are characteristic pathological features of human neuro-inflammatory and neurodegenerative disorders of the CNS that include AD (Calsolaro and Edison, 2016; Andreeva et al., 2017). What is generally not appreciated is that a major secretory product of the GI tract microbiome is amyloid, and that the life-long contribution of microbial amyloid to CNS pathophysiology can be very substantial. “Amyloid” is a generic term for any aggregated, insoluble, lipoprotein-enriched deposit that exhibits β-pleated sheet structures oriented perpendicular to the fibrillar axis (Lukiw, 2012; Clark and Vissel, 2015; Lim et al., 2015; Andreeva et al., 2017; Bolós et al., 2017). The potential for amyloid formation is surprisingly high in almost all proteins; a major factor for amyloid formation is the presence within proteins of primary amino acid sequences that can form a tight, self-complementary interface with an identical segment, thus permitting the cooperative formation of a steric zipper. Two self-complementary beta-sheets form the backbone of the amyloid fibril (Goldschmidt et al., 2010; Buxbaum and Linke, 2012; Andreeva et al., 2017). The characterization of the “amylome,” a categorization of amino acid sequences that possess self-complementary interfaces and high fiber-forming propensity has improved our understanding of the capability of different proteins to generate amyloid (Goldschmidt et al., 2010; Lukiw, 2012; Andreeva et al., 2017). The progressive generation and aggregation of amyloids contribute to “dense-deposit” disease; the pathogenesis of diseases that accumulate amyloid, including AD, all involve prominent inflammatory responses at sites of amyloid deposition—these accumulations are often mediated by microglial cells, the “resident immune cells” of the CNS. Interestingly, most microbial species, including fungi and bacteria, secrete self-associating and strongly amyloidogenic lipoproteins (Hill et al., 2014; Syed and Boles, 2014; Schwartz et al., 2016). For instance, amyloids are associated with fungal surface-structures and the recent observation of amyloidogenic fungal proteins and diffuse mycoses in the blood of AD patients suggest that chronic fungal infection over the course of aging may increase AD risk (Alonso et al., 2014; Hill et al., 2014). Of further relevance is that: (i) Aβ42 peptide monomers, dimers, oligomers and fibrils each induce patterns of pro-inflammatory gene signaling typical of the classical microglial-mediated innate-immune and inflammatory response induced by infectious agents such as bacterial LPS (Ferrera et al., 2014; Calsolaro and Edison, 2016; Lukiw, 2016; Andreeva et al., 2017); (ii) the presence of bacterial LPS or endotoxin/exotoxin-mediated inflammatory signaling strongly contributes to amyloid neurotoxicity (Lee et al., 2008; Asti and Gioglio, 2014; Zhao and Lukiw, 2015; Zhao et al., 2016); (iii) AD amyloids, like prion amyloids, once formed, may induce a self-perpetuating process leading to amplification, aggregation, and spreading of pathological aggregates (Le et al., 2014); and (iv) recently it has been shown that Aβ42 peptide fibrillogenesis is strongly potentiated by soluble bacterial exudates and viruses such as HSV-1, suggesting the contribution of microbial-sourced factors and/or infectious events to amyloidogenesis, a distinguishing feature of the AD neuropathology (Hill et al., 2014; Stilling et al., 2014; Zhao et al., 2015; Russo et al., 2017).

## Small non-coding RNA (sncRNA)

While the secretion of proteins, lipids, and nucleic acids (both RNA and DNA) from neural cells into the extracellular space is a commonly recognized phenomenon in neurobiology, the secretion of small non-coding RNA (sncRNA) from microbial cells into the GI tract has only been very recently characterized (Ghosal et al., 2015; Lukiw, 2016; Ghosal, 2017). Employing multi-component secretion systems, sncRNAs may be exuded from bacteria as separate entities, or more commonly, contained within lipid spheres or outer membrane vesicles (OMVs; Ghosal et al., 2015; unpublished observations). A major fraction of all secreted extracellular RNAs are sncRNAs in the size range of 15–40 nucleotides derived from specific intracellular bacterial RNAs. These sncRNAs have been speculated to be involved in immune-evasion, intra-species communication, in inter-kingdom genetic exchanges, pathogenicity and/or microbiome-host signaling; indeed protein-, lipid-, and nucleic acid-containing OMVs released by GI tract Gram-negative bacteria can be intensely pro-inflammatory, pathogenic or even lethal to the host (Zhao and Lukiw, 2015; Ghosal, 2017; Lukiw and Rogaev, 2017; unpublished observations). Several important questions remain to be answered: (i) do secreted sncRNAs play any role in microbiome survival, immune evasion and/or antibiotic resistance? (ii) how do GI tract microbes promote and organize the regulation of sncRNA trafficking (iii) how are bacterial sncRNAs transported across bacterial membranes and subsequently released into the extracellular space? (iv) how are the sncRNAs selected and packaged for export? and (v) are there differences in secreted sncRNA profiles between pathogenic and non-pathogenic bacteria and/or between healthy and diseased states of the host? Further investigations in the field of extracellular bacterial sncRNAs are clearly needed to shed light on their potential role as mediators of microbiome-host signaling and intercellular communication. By studying bacterial secreted sncRNA patterns, we may be able to further advance our understanding of the complex interactions that exist between humans and their GI tract microbiome and design, perhaps through dietary manipulation, highly effective intervention strategies that could improve and optimize human neurological health.

## Thanatomicrobiome

Evidence for the immense biophysiological efforts in keeping the GI tract microbiome contained within GI tract compartments and from expansion beyond its normal niche, comes from analysis of the human microbiome at the time of death. Very little data are available concerning what happens to the microbiome when a human host dies—in a healthy adult, most internal organs such as the spleen, liver, heart, and brain are generally devoid of microbes because the innate-immune system or other microbial components keeps them in check. After death, however, the generation of ATP ceases, the innate-immune system falters and microbes proliferate throughout the body; this has recently been shown to begin in the ileocecal area of the GI tract, spreading to the liver and spleen, and continuing to the heart and brain (Alan and Sarah, 2012; Can et al., 2014; Clement et al., 2016; Javan et al., 2016). Still evolving concepts of what happens to GI tract microbiome speciation and complexity at the time of death are currently being researched. Indeed the thanatomicrobiome (thanatos, Greek for death) is a relatively new designation defined as the composition and organization of the GI tract microbiome and other microbial communities following cessation of all life activities (Clement et al., 2016; Javan et al., 2016). Recent studies so far underscore the fact that in the GI tract microbiome there is a constant struggle to contain GI tract microbiome integrity and regulate specific bacterial abundance and complexity (Clement et al., 2016; Javan et al., 2016). Ongoing work from temporal studies on the thanatomicrobiome across defined post-mortem intervals (PMI) further indicate (i) that the majority of the microbes within the human body and those which propagate most rapidly at the time of death are the obligate anaerobes that begin to non-randomly proliferate from the GI tract continuing throughout the human organs over the PMI (Javan et al., 2016); and (ii) that comprehensive knowledge of the number and abundance of each organ's microbial signature could be useful to forensic microbiologists as a new source of data for estimating PMI. These data combined with nucleic acid sequencing and bioinformatics would also be invaluable in aiding researchers who use post-mortem tissues in their research work and in forensic criminology, microbial speciation and the study of microbiome-host genetics in the later stages of life.

## Concluding remarks

**Figure 2:**
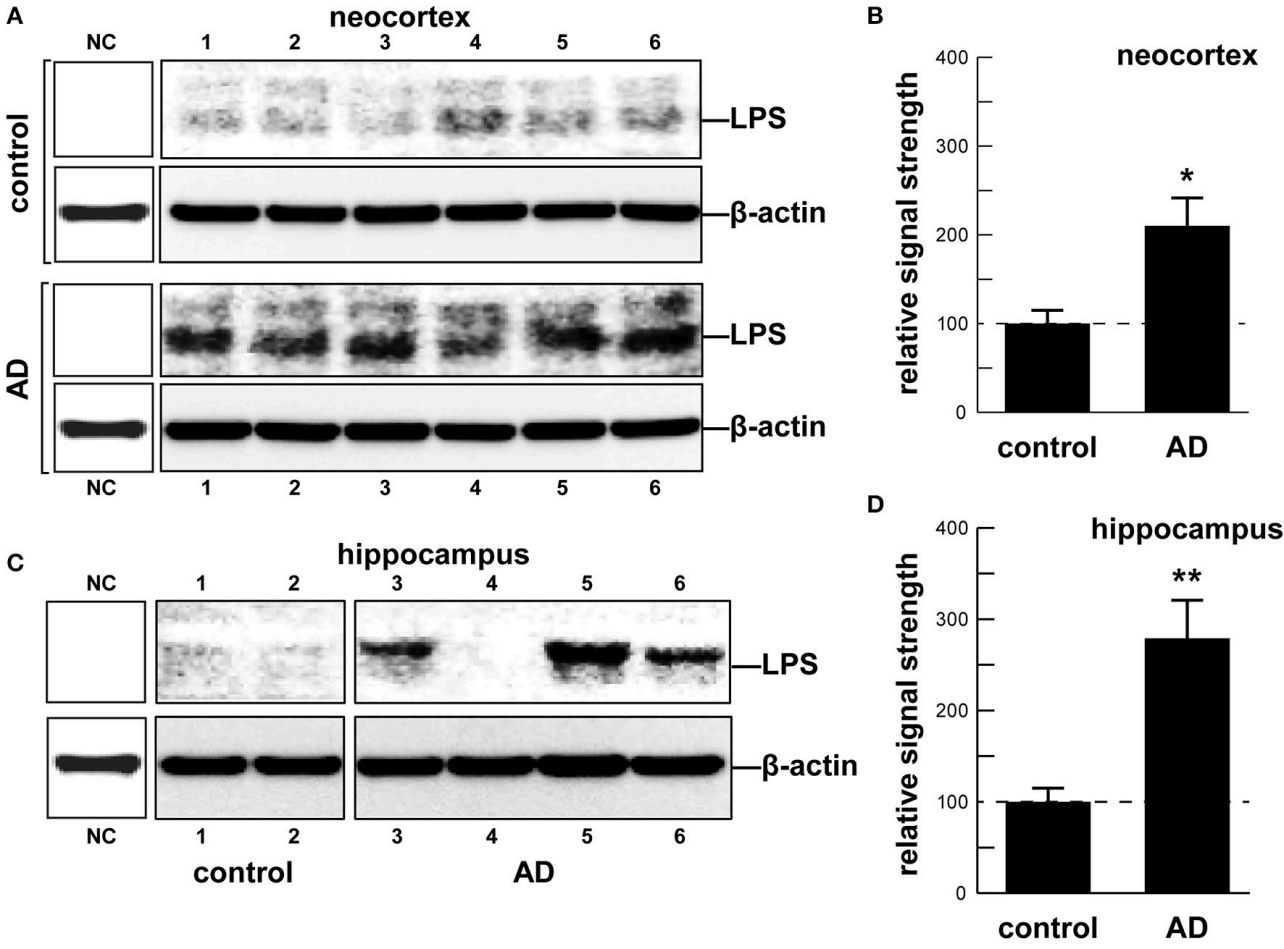
**(A)** human brain temporal lobe neocortex [*N* = 6 control and 6 sporadic AD cases; quantified in (**B)**]; and **(C)** hippocampus (*N* = 2 control and *N* = 4 AD cases; quantified in **(D)**] were analyzed for LPS against β-actin abundance in the same sample (using anti-*E. coli* LPS; cat # ab35654 from Abcam, Cambridge UK and anti-β-actin cat # 3700 from Cell Signaling, Danvers MA, USA) using Western analysis as previously described by our group (Bhattacharjee et al., 2016; Zhao et al., 2016); all AD and control tissues were analyzed in a RNA-analysis clean room facility; all control and AD tissues were age- and gender-matched; there were no significant differences between the age (control 72.9 ± 8.1 years, AD 74.2 ± 9.1 years), gender (all female), PMI (all tissues 3.5 h post-mortem or less), RNA quality or RNA yield between each of the two groups; LPS abundance was found to be on average over two-fold as abundant in AD when compared to age-, gender, and PMI-matched control neocortex in 6 of 6 cases; LPS was found to be on average three-fold as abundant in AD when compared to age-, gender, and PMI-matched control hippocampus in 3 of 4 cases; some advanced AD hippocampal samples exhibited up to a 26-fold increase in LPS over age-matched controls (**C**, LPS in control lane 2 vs. AD lane 5); because one major source of LPS are Gram-negative bacteria of the human GI tract (predominantly *B. fragilis* and *E. coli*), this suggests that *in vivo* intensely pro-inflammatory LPS species may be able to “leak” through at least two major biophysiological barriers—the GI tract barrier and the BBB—to access brain compartments (see Devier et al., 2015; Halmer et al., 2015; Choi et al., 2016; Minter et al., 2016b; Montagne et al., 2016; Richards et al., 2016; Soenen et al., 2016; van de Haar et al., 2016; Zhan and Davies, 2016; Zhao et al., 2016; Varatharaj and Galea, 2017). Unpublished work from this laboratory further indicates the positive detection of LPS in 36 of 36 AD tissues sampled from the superior temporal lobe neocortex in aged individuals (age range 66–79 yr; see Table 1 in Cui et al., 2010). Another recent investigation reports the finding of LPS in gray matter (temporal lobe) and white matter (frontal lobe) of the AD brain (Zhan et al., 2016). Together these data also suggest that neurotoxic cocktails secreted by multiple GI tract microbes or other microbial species (Figure 1) may have considerable potential to support intense pro-inflammatory signaling within the CNS especially over the course of aging when barriers become more “leaky” (Hill and Lukiw, 2015; Keaney and Campbell, 2015; Montagne et al., 2015; Choi et al., 2016; Köhler et al., 2016; Minter et al., 2016a,b; Richards et al., 2016; van de Haar et al., 2016; Zhan and Davies, 2016; Varatharaj and Galea, 2017); **(B)** and **(D)** represent the mean plus one standard deviation of that mean; ^*^p < 0.05, ^**^p < 0.01 ANOVA; NC, negative control using a control murine brain extract (strain C57BL/6J); in **(B)** and **(D)** a dashed horizontal line at 100 is included for ease of comparison.

In summary, the human GI tract constitutes the largest repository of the human microbiome, and its impact on human neurological aging, health and disease is becoming increasingly appreciated. Consisting of about ~4 × 10^13^ microorganisms, the human GI tract microbiome forms a highly complex, symbiotic and dynamic ecosystem within the host and dietary factors and host genetics appear to have a strong influence on microbial abundance, speciation and complexity, and their ability to influence CNS functions (Foster et al., 2016; Li et al., 2016; Richards et al., 2016; Brandscheid et al., 2017; Tremlett et al., 2017). We sincerely hope that this “Perspectives” article has effectively highlighted recent findings on microbial-derived endotoxins, exotoxins, LOSs and LPSs, amyloids and sncRNAs and has stimulated interest in the potential contribution of these neurotoxic and pro-inflammatory microbial exudates to age-related inflammatory neurodegeneration, amyloidogenesis, and AD-relevant pathology (Figure 1). Taken together, these current observations and recent data advance at least seven areas in our understanding of the role of the GI tract microbiome in age-related neurological diseases associated with progressive, inflammatory neurodegeneration of the human brain: (i) that the GI tract microbiome are a potent source of neurotoxic species that are abundantly secreted by multiple Gram-negative bacilli in the gut (*B. fragilis, E. coli*, and others); (ii) that bacterial LPS are readily detectable in the neocortex and hippocampus of the AD brain, and at significantly higher abundance in AD than controls, indicating that LPS may be able to transit physiological barriers to access CNS compartments (Figure 2); (iii) that the transit of highly pro-inflammatory neurotoxins such as LPS across compromised GI tract and blood-brain barriers underscore the critical roles of cellular adhesion structures in allowing passage of noxious molecules from the GI tract into the systemic circulation and CNS (Montagne et al., 2016; Soenen et al., 2016; van de Haar et al., 2016); (iv) that extremely complex mixtures of neurotoxins may be generated by either single microbes or by combinations of bacilli that constitute the GI tract microbiome (Figure 1); (v) that biophysical, gastrointestinal, and neurobiological barriers that may become more “leaky” with aging again underscore the important role of intact membrane barriers in moderating systemic and CNS inflammation and immune-mediated inflammatory disease (Hill and Lukiw, 2015; Keaney and Campbell, 2015; Montagne et al., 2015; Choi et al., 2016; Köhler et al., 2016; Minter et al., 2016a; Richards et al., 2016; van de Haar et al., 2016; Zhan and Davies, 2016; Varatharaj and Galea, 2017); (vi) that bacterial complexity, neurotoxin abundance, speciation, and complexity in the CSF, blood serum or in brain tissues may be useful for the diagnosis of AD (Zhao et al., 2015; Soenen et al., 2016); and (vii) that studies on the thanatomicrobiome should be useful for a clearer understanding of the neuro- and micro-biological processes in operation over the PMI that should be useful in scientific research that utilizes post-mortem tissues in basic research, in forensic applications, in criminology and in the more accurate diagnosis of neurological disease (Clement et al., 2016; Javan et al., 2016). While one other recent investigation reported the detection of LPS in gray matter (temporal lobe) and white matter (frontal lobe) in AD (Zhan et al., 2016), here for the first time we report the detection of bacterial LPS in brain lysates from AD hippocampus, an anatomical region of the AD brain that develops the earliest and most profound neuropathology. Some advanced AD hippocampal patients exhibited up to a 26-fold increase in LPS over age-matched controls. Lastly, more research into the intriguing field of human GI tract microbiome-host interaction and its potential contributory role to human aging, neurological health and disease is clearly needed. The study of these symbiotic prokaryotic and eukaryotic divisions, their evolution and their intriguing interrelationships, genetic interactions and associations in future work should be useful in expanding our understanding of microbiome-host interplay and control in the initiation, development, propagation, and diagnosis of human neurological disorders in which microbial involvement appears to play some contributory or even deterministic role.

## Author contributions

YZ and VJ analyzed brains for LPS content; WL compiled and analyzed the data and wrote the paper.

### Conflict of interest statement

The authors declare that the research was conducted in the absence of any commercial or financial relationships that could be construed as a potential conflict of interest.
